# Screening the Chemical Composition, Antioxidant Activity, and Toxicity of Ten Species of Non‐Conventional Food Plants From Southern Brazil

**DOI:** 10.1002/cbdv.202503521

**Published:** 2026-04-15

**Authors:** Luana Vanessa Peretti Minello, Paola Dutra da Rosa, Sabrina dos Santos Cassol, Luciana Bavaresco Andrade Touguinha, Cátia Santos Branco, Valdirene Camatti Sartori

**Affiliations:** ^1^ Organic Agriculture Laboratory Biotechnology Institute University of Caxias do Sul Francisco Getúlio Vargas Caxias do Sul RS Brazil; ^2^ Center For Innovation and Development in Sustainable Agriculture Biotechnology Institute University of Caxias do Sul Francisco Getúlio Vargas Caxias do Sul RS Brazil; ^3^ Plant Stress Laboratory Institute of Botany Federal University of Pelotas, Avenida Eliseu Maciel Capão do Leão RS Brazil; ^4^ Plant Biotechnology Laboratory Biotechnology Institute University of Caxias do Sul Francisco Getúlio Vargas Caxias do Sul RS Brazil; ^5^ Oxidative Stress and Antioxidants Laboratory Biotechnology Institute University of Caxias do Sul Francisco Getúlio Vargas Caxias do Sul RS Brazil

**Keywords:** phytochemistry, redox capacity, secondary metabolites, toxicity, wild edible plants

## Abstract

Non‐conventional food plants (NCFPs) are resilient, adaptable species with nutritional qualities. Brazil has the world's greatest biodiversity in these species. Despite evidence of their potential for sustainable nutrition, there are few related studies. Therefore, this study aimed to evaluate the antioxidant capacity, total polyphenol content, main compounds, and in vivo toxicity of ten NCFPs from southern Brazil. The results showed that the NCFP extracts presented considerable variability in antioxidant activity. Notably, ABTS scavenging ranged from 21.70% ± 0.84% to 99.54% ± 0.01%, whereas DPPH values ranged from 56.88 ± 2.32% to 95.10 ± 1.09%. Total phenolic content varied between 24.43 ± 1.35 and 260.38 ± 12.92 mg GAE g^−^^1^. HPLC‐UV analysis identified gallic acid as the main phenolic compound. Epicatechin, rutin, naringin, and hesperidin were also identified. All extracts showed no toxicity in the *A. salina* assay at concentrations up to 5000 µg/mL after 24 h of exposure. The results of this study highlight the high antioxidant activity and the presence of essential phenolic compounds in NCFP, highlighting its potential health benefits as protective agents against oxidative damage, as well as its social and economic relevance.

## Graphical abstract

This study found that the ten unconventional food plants from southern Brazil studied exhibited high antioxidant activity, a diversity of phenolic compounds, and no toxicity in vivo. These data demonstrate that these plants have promising benefits for human health and may be a potential protective agent against oxidative damage.

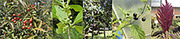

## 1 Introduction

Plants are fundamental to human existence, due to their practical, cultural, nutritional, and medicinal properties [1, 2]. Current data indicate that only 103 species make up 90% of the global food supply, leading to the marginalization of more rustic and underutilized species [1, 3, 4, 5]. This narrowing of food diversity has contributed to the neglect of numerous edible species traditionally used by local populations, commonly referred to as non‐conventional food plants (NCFPs) [6, 7, 8, 9, 10]. It should be considered that the NCFPS which have importance and impact as possible sources of eliminating hunger and world poverty, in addition to implementing an increase in critical nutritional profiles in the human diet [1, 11, 12, 13].

NCFPs are regarded as hardy, highly adaptable, and phenotypically plastic species. Many are endemic or native and, unlike cultivated crops, have not been subjected to genetic improvement [5, 13, 14, 15]. These characteristics confer tolerance to adverse environmental conditions and highlight their potential contribution to resilient and sustainable food systems [16, 17, 18, 19]. Nevertheless, their use remains largely restricted to small communities and indigenous groups, mainly due to limited scientific validation regarding their nutritional composition, bioactive profiles, and safety [2, 11, 15].

It is worth noting that some of these plants have been used for many years by local populations, who are well acquainted with their properties. Expanding research and utilizing advanced tools for accurate identification, composition analysis, and toxicology can enhance our understanding of the economic and social value of these plants [11].

Brazil is the country with the most remarkable plant biodiversity on the planet, with a high level of endemic species, many of which are considered NCFP [20, 21]. Unfortunately, due to the extensive use of monocultures many of these have been marginalized even though many are known to contain high levels of nutritionally relevant and biologically active compounds [14, 22]. Previous studies indicate that NCFPs are promising sources of minerals, dietary fiber, vitamins (tocopherol and ascorbic acid), and antioxidant compounds, including phenolics, flavonoids, anthocyanins, and carotenoids [5, 11, 15, 23, 24, 25, 26, 27]. These metabolites are associated with antioxidant activity and protective effects against oxidative stress‐related disorders [28, 29, 30, 31]. However, most available studies are fragmented, species‐specific, and often lack integrated assessments combining chemical characterization, antioxidant potential, and toxicological safety.

In this context, a knowledge gap persists regarding the comprehensive evaluation of the bioactive composition, antioxidant capacity, and in vivo safety of products that can be derived from these plants. The ten NCFP species investigated in this study were selected based on their occurrence in southern Brazil, documented traditional consumption by local communities, and availability during the harvest period, in addition to being understudied using a standardized and integrated experimental approach. Their selection, therefore, reflects both their ethnobotanical relevance and the need to generate systematic chemical and biological data to support their scientific validation and potential valorization. Thus, the present study aimed to evaluate the antioxidant capacity, total polyphenol content, main bioactive compounds, and in vivo toxicity of extracts obtained from ten native NCFP species from this region, providing scientific evidence to support their safe use, and potential valorization as functional food resources.

## 2 Results and Discussion

### 2.1 Determination of the Antioxidant Capacity of Extracts

The evaluation of antioxidant capacity using the DPPH and ABTS methods revealed significant variation in the total values among the fruits; notably, the most significant variation was observed in the ABTS assay with values ranging from 21.70% ± 0.84% to 99.54% ± 0.01%. In contrast, DPPH values ranged from 56.88% ± 2.32% to 95.10% ± 1.09%. The plant that presented the lowest rate of DPPH inhibition was *Citharexylum solanaceum* (56.88% ± 2.32%), while the fruit with the highest DPPH antioxidant capacity was *Zanthoxylum rhoifolium* (95.10% ± 1.09%), followed by *Ilex theezans* (88.98% ± 2.88%) and *Myrsine parvula* (83.50% ± 0.46%). For the ABTS radical the lowest scavenging activity was *Ilex theezans* (21.70% ± 0.84%), and the highest was *Myrcia oblongata* (99.54% ± 0.01%), followed by *Vitex megapotamica* (90.39% ± 1.08%) and *Zanthoxylum rhoifolium* (87.17% ± 0.10%). These results are presented in Table 1.

**TABLE 1:** Antioxidant activity of fruit extracts from non‐conventional food plants (NCFPs), determined by DPPH (% inhibition), ABTS (% inhibition) radical scavenging assays, and total phenolic content (TPC) measured by the Folin–Ciocalteu method (mg GAE g^−^^1^).

Scientific name	DPPH (% inhibition)	ABTS (% inhibition)	TPC (mg GAE g^−1^)
*1‐ Citharexylum solanaceum*	56.88 ± 2.32 g	58.86 ± 1.86 d	61.60 ± 4.38 d
*2‐ Ilex theezans*	88.98 ± 2.88 b	21.70 ± 0.84 f	35.90 ± 2.40 e
*3‐ Myrceugenia miersiana*	74.60 ± 2.05 ef	25.26 ± 1.96 f	79.79 ± 0.79 d
*4‐ Myrcia oblongata*	78.49 ± 0.93 de	99.54 ± 0.01 a	24.43 ± 1.35 e
*5‐ Myrsine parvula*	83.50 ± 0.46 c	62.54 ± 2.50 d	147.23 ± 1.55 b
*6‐ Psidium cattleyanum*	81.99 ± 1.19 cd	82.93 ± 0.96 c	71.70 ± 0.14 d
*7‐ Solanum pseudoquina*	82.98 ± 0.83 c	57.92 ± 1.80 d	121.53 ± 2.62 c
*8‐ Tournefortia paniculata*	82.78 ± 0.54 cd	42.02 ± 2.66 e	154.68 ± 4.22 b
*9‐ Vitex megapotamica*	73.95 ± 0.54 f	90.39 ± 1.08 b	260.38 ± 12.92 a
*10‐ Zanthoxylum rhoifolium*	95.10 ± 1.09 a	87.17 ± 0.10 bc	141.93 ± 9.16 b

*Note*: Values are expressed as mean ± SD (*n* = 5). Within each column, different lowercase letters indicate statistically significant differences according to one‐way ANOVA followed by Tukey's multiple comparison test (*p* ≤ 0.05); identical letters denote no significant difference.

The antioxidant capacity, largely determined by plant secondary metabolism and chemical composition, is associated with the ability to capture and neutralize free radicals, thereby contributing to the maintenance of cellular redox homeostasis [32]. Several mechanisms are involved in oxidative processes, including single electron transfer (SET) and hydrogen atom transfer (HAT) pathways [33]. A range of methods can be employed to evaluate antioxidant capacity, notably colorimetric assays such as the DPPH assay, which primarily measures radical scavenging activity in organic media, and the ABTS assay, which can be applied in both aqueous and organic systems. Moreover, the Folin‐Ciocalteu method, which is based on the reduction of samples with phenolic compounds in an alkaline state can also be highlighted [34, 35].

The differences observed between DPPH and ABTS results among the evaluated fruits may be explained by the distinct physicochemical characteristics of the radicals and their interaction with phenolic constituents. The DPPH• radical, being sterically hindered and typically dissolved in organic solvents, may show greater sensitivity to compounds capable of efficient hydrogen atom or electron donation under less polar conditions. In contrast, the ABTS•^+^ radical cation is more accessible and reactive in aqueous environments, allowing interaction with a broader range of hydrophilic antioxidants [36]. Therefore, variations in antioxidant performance among species likely reflect differences in phenolic structure, degree of hydroxylation, conjugation, and possible glycosylation patterns, which influence both solubility and reaction kinetics in each assay system.

These results indicate marked interspecific variability among the evaluated NCFPs, suggesting that antioxidant performance is strongly dependent on the intrinsic phytochemical composition of each species. *Zanthoxylum rhoifolium* exhibited consistently high activity in both DPPH and ABTS assays, suggesting the presence of phenolic constituents capable of acting efficiently through both SET and HAT mechanisms across different reaction media [33]. In contrast, *Ilex theezans* showed high activity in the DPPH assay but markedly lower activity in ABTS, indicating that its antioxidant response may be more effective under the organic conditions of the DPPH system and possibly influenced by structural features that affect solubility, steric accessibility, or reaction kinetics in aqueous environments.

The strong ABTS activity observed for *Myrcia oblongata* and *Vitex megapotamica*, combined with their moderate DPPH radical scavenging activity (78.49% ± 0.93% and 73.95% ± 0.54%, respectively), further highlights that antioxidant capacity is not uniform across assays and likely reflects differences in phenolic composition, degree of hydroxylation, conjugation, and glycosylation patterns.

### 2.2 Total Phenolic Compound Content (TPC) of Extracts (Folin‐Ciocalteu Analysis)

Data related to the Folin‐Ciocalteu are described in Table 1. The average ​​of phenolic content ranged between 24.43 ± 1.35 and 260.38 ± 12.92 mg GAE g^−^^1^, with substantial variability among the analyzed samples. The plant with the lowest phenolic content was observed in *Myrcia oblongata* (24.43 ± 1.35 mg GAE g^−^^1^), whereas the highest value was detected in the fruit extract of *Vitex megapotamica* (260.38 ± 12.92 mg GAE g^−^^1^). These results differ from those reported for *Echinacea* species, in which total phenolic contents ranging from 21.15 to 78.34 mg GAE g^−^^1^ have been described, depending on the species analyzed [34]. Similarly, extracts of *Citrullus colocynthis* L., *Cucumis sativus* L., and *Momordica charantia* L. have been reported to exhibit lower phenolic contents (50.87, 56.58, and 42.36 mg GAE g^−^^1^, respectively), as determined by the Folin–Ciocalteu assay [34].

### 2.3 Antioxidant Activity and Its Relationship With Phenolic Compounds

The extract of *Zanthoxylum rhoifolium* presented the best overall antioxidant activity, as well as a high concentration of total polyphenols. In contrast, the extract of *Citharexylum solanaceum* presented generally reduced antioxidant capacity and low levels of total polyphenols.

When the data from the assays of the antioxidant activities of DPPH, ABTS, and the content of total phenolic compounds were evaluated through Pearson's Correlation (Table 1), it was possible to verify a null correlation between DPPH and ABTS (*r* = 0.15; *p* ≤ 0.001). The correlation between ABTS and total polyphenols was very weak (*r* = 0.254; *p* ≤ 0.001). However, the data showed a strong positive correlation between DPPH and total polyphenols (*r* = 0.82; *p* ≤ 0.001), indicating that phenolic compounds substantially contribute to the antioxidant capacity measured by the DPPH assay. These findings demonstrate that the strength of the relationship between antioxidant activity and phenolic content depends on the analytical method employed.

Previous reports in some genera of the Lamiaceae family have described a strong positive correlation between DPPH and ABTS assays (*r* = 0.669) [37]. However, the absence of such correlation in the present study suggests that the antioxidant behavior of these fruit extracts is not uniform across assay systems and may reflect differences in chemical composition and compound reactivity.

The strong association between DPPH activity and total phenolic content supports the contribution of phenolic hydroxyl groups as effective hydrogen or electron donors in this system. Conversely, the weak correlation observed for ABTS indicates that radical scavenging in this assay may involve additional antioxidant constituents beyond phenolic compounds. Together, these results confirm that DPPH and ABTS assays provide complementary, rather than interchangeable, information regarding antioxidant potential in chemically complex fruit extracts.

### 2.4 Quantitative Analysis of Phenolic Compounds by HPLC‐UV

In Table 2, HPLC revealed gallic acid as the primary compound in all evaluated extracts as the predominant phenolic compound. *Myrceugenia miersiana* presented the highest gallic acid amounts (110.29 µg.mL^−1^), while *Zanthoxylum rhoifolium* showed the lowest (23.88 µg.mL^−1^). *Ilex theezans* was the only one in which other phenolic compounds were identified (epicatechin, rutin, naringin, and hesperidin). These results suggest that this plant may have broader potential application, owing to the greater diversity of its bioactive compounds.

**TABLE 2:** Identification and quantification of phenolic compounds determined by high‐performance liquid chromatography with UV detection (HPLC–UV).

Scientific name	Gallic acid (µg mL^−1^)	Epicatechin (µg mL^−1^)	Rutin (µg mL^−1^)	Naringin (µg mL^−1^)	Hesperidin (µg mL^−1^)
*1‐ Citharexylum solanaceum*	70.4	ND	ND	ND	ND
*2‐ Ilex theezans*	42.93	32.71	46.74	33.73	105.64
*3‐ Myrceugenia miersiana*	110.29	ND	ND	ND	ND
*4‐ Myrcia oblongata*	32.87	ND	ND	ND	ND
*5‐ Myrsine parvula*	67.8	ND	ND	ND	ND
*6‐ Psidium cattleyanum*	53.92	ND	ND	ND	ND
*7‐ Solanum pseudoquina*	56.81	ND	ND	ND	ND
*8‐ Tournefortia paniculata*	106.36	ND	ND	ND	ND
*9‐ Vitex megapotamica*	30.85	ND	ND	ND	ND
*10‐ Zanthoxylum rhoifolium*	23.88	ND	ND	ND	ND

*Note*: Results are expressed as µg mL^−^^1^ (mean ± SD, *n* = 5). ND: not detected. Sample size: 10 plants.

This study identified gallic acid in all the plants examined. Gallic acid is a phenolic compound known for its anti‐inflammatory, anti‐lipidemic, and anticancer properties. Previous research has demonstrated its antitumor potential by inhibiting migration and metastasis pathways, inducing apoptosis and cell cycle arrest, and downregulating oncogenic expression [38, 39].

Our data also identified the presence of epicatechin, rutin, naringin, and hesperidin in the *Ilex theezans*. Epicatechin is a monomeric flavanol with a direct effect on mitochondrial function and biogenesis [40]. Furthermore, its tumor‐inhibitory effects were previously demonstrated in *Camellia sinensis* [41]. This compound has numerous other beneficial functions, including a positive influence on primary hemostasis, coagulation, and fibrinolysis [42]. This flavonoid bioactivity may influence cellular oxidant production and redox signaling by regulating pathways of nuclear factor E2‐related factor 2 (Nrf2), which enhances antioxidant defenses, and the nuclear factor kappa beta (NF‐kB), which controls the expression of oxidant‐generating enzymes [43]. Rutin is a flavonoid whose pharmaceutical and nutraceutical properties remain underexplored. However, studies show that, when combined with alpha‐tocopherol, it significantly improves venous tone, capacity, and distension, and enhances fibrinolysis through increased tissue plasminogen activator activity [44]. Citrus flavonoids are crucial herbal additives that have a broad spectrum of organic activities and are very commonly found in NCFP. Thus, naringin is a nutritional flavonoid glycoside that is effective in treating certain chronic disorders associated with aging. This compound is known for its anti‐inflammatory and antitumor effects. In addition, studies have demonstrated its efficient action in the treatment of asthma, hyperlipidemia, diabetes, cancer, hyperthyroidism, bone and cartilage diseases, and in combating sepsis [45, 46]. Hesperidin is a glycosidic flavonoid found mainly in citrus fruits that has high antioxidant, antibacterial, anti‐inflammatory, and antitumor actions [47]. Recent studies have reported its essential role as a neuroprotective agent in neurological diseases such as Parkinson's, Alzheimer's, Huntington's, and multiple sclerosis [48, 49].

In studies using HPLC coupled to UV or photodiode array detection, several phenolic acids and flavonoids have been identified in leaves of *Ilex* species, including *Ilex theezans*, such as caffeoyl derivatives, chlorogenic acids, quercetin, rutin, and kaempferol [50]. Among these compounds, only rutin corroborates the present findings. It is important to note that most available studies involve different plant organs and extraction conditions, which may explain the differences in phenolic profiles observed.

The nutraceutical potential of NCFPs has also been reported for other species, including *Chenopodium album*, *Bacopa monnieri*, *Euterpe oleracea*, *Talinum triangulare*, and *Bidens bipinnata*, which are often highlighted due to their antioxidant capacity and diverse phenolic composition [5, 11, 51, 52, 53, 54, 55, 56, 57, 58, 59, 60, 61, 62, 63, 64]. However, as these species were not included in the experimental design of the present study and were not analyzed by HPLC, they are mentioned here only to contextualize the broader relevance of NCFPs, without direct comparison to the results obtained.

Thus, it is clear that despite the diversity of compounds found in the different NCFPs, all of them present high antioxidant activities and essential phenolic compounds that are beneficial to human health and protective agents against oxidative damage. Given the pharmaceutical importance and useful properties of NCFP proven by this research and countless others that are delving deeper into this issue, the need arises to verify the toxicity of these plants. Therefore, some authors emphasize that toxicity studies should be conducted in conjunction with pharmacological evaluations to ensure the safe use of these plants as potential therapeutic agents [65].

It is important to emphasize that the present HPLC–UV analysis was targeted and limited to phenolic standards commercially available at the time of the study. Therefore, the quantified compounds represent selected phenolic markers rather than a comprehensive characterization of the extracts. Given the chemical complexity of plant matrices, other phenolic subclasses and structurally related metabolites may be present but were not identified under the applied analytical conditions. Future investigations employing more comprehensive analytical platforms, such as HPLC–DAD–MS or LC–MS/MS, are necessary to achieve broader metabolite profiling and to elucidate minor constituents that may contribute to the observed biological activities.

### 2.5 Evaluation of in Vivo Toxicity in *Artemia salina*

All extracts obtained through a green process and without the addition of organic solvents were tested at concentrations up to 5000 µg/mL for toxicity evaluation over a standard 24‐h period. The data clearly showed no nauplii mortality, indicating that all NCFP extracts were safe in the *A. salina* assay at the concentrations tested. Because no mortality occurred under the experimental conditions employed, the median lethal dose (LD_50_) could not be determined. Letality test in *Artemia* sp is considered the most appropriate and advantageous way to examine the biological activities of different plant extracts, since it is regarded as a safe, economical, simple, fast, reliable, and comprehensive method. It can be used to detect general toxicity in a broad spectrum of natural products [66]. In the present study, all evaluated NCFP extracts exhibited a favorable preliminary safety profile in this bioassay, supporting their further investigation using more specific toxicological models.

### 2.6 General Assessments

It is essential to acknowledge the study's limitations in precisely quantifying DPPH, ABTS, and total polyphenols, as well as potential variations in chemical composition (thus physicochemical parameters such as pH, titratable acidity, or fruit color were not measured) due to extract preservation methods and differences in fruit ripening stages. These factors, as already discussed by other authors, may affect the antioxidant activity and the phytochemicals present [31]. It is also worth noting that, like all toxicity tests, the *A. salina* model has limitations, such as the solubility of the toxic agent in the saline environment in which the animals are confined, which can generate false positives [67].

Regarding toxicity evaluation, although the *A. salina* model is widely accepted for preliminary screening, it has inherent limitations, including dependence on compound solubility and bioavailability in saline medium, which may influence test sensitivity and lead to under‐ or overestimation of toxicity [67]. Therefore, absence of lethality in this assay does not preclude the need for further in vitro and in vivo toxicological assessments.

Despite these limitations, the combined chemical and biological data demonstrate that the investigated NCFPs are characterized by significant antioxidant activity, a diverse phenolic composition, and absence of acute toxicity in the *A. salina* model. These findings reinforce the relevance of these species as potential sources of bioactive compounds and justify further pharmacological, toxicological, and nutraceutical investigations to substantiate their functional and dietary applications.

## 3 Conclusion

This study clearly and precisely concluded that the ten NCFP from Southern Brazil present high antioxidant activity, a diversity of phenolic compounds, and an absence of in vivo toxicity. The combination of spectrophotometric assays and HPLC‐UV analysis confirmed that phenolic constituents substantially contribute to the observed radical scavenging activity, although assay‐dependent differences were evident.

The absence of acute toxicity in the brine shrimp bioassay, together with the significant antioxidant potential, underscores the relevance of these NCFPs as promising sources of bioactive metabolites. These findings support their prospective application as natural antioxidant agents in pharmaceutical and nutraceutical formulations. Nonetheless, further studies involving in vitro cellular systems and in vivo models are required to confirm biological efficacy, bioavailability, and safety under physiological conditions.

## 4 Experimental Section

### 4.1 Chemicals and Reagents

All chemicals and reagents used in this study were of analytical or HPLC grade. Gallic acid, quercetin, rutin, caffeic acid, and other phenolic standards were purchased from Sigma–Aldrich (St. Louis, MO, USA). Folin–Ciocalteu reagent, 2,2‐diphenyl‐1‐picrylhydrazyl (DPPH), and 2,2′‐azino‐bis (3‐ethylbenzothiazoline‐6‐sulfonic acid) (ABTS) were obtained from Sigma–Aldrich (St. Louis, MO, USA). Methanol and acetonitrile (HPLC grade) were acquired from Merck (Darmstadt, Germany), and formic acid from Vetec (Rio de Janeiro, Brazil). Sodium carbonate and potassium persulfate were purchased from Synth (Diadema, SP, Brazil). Ultrapure water was obtained from a Milli‐Q purification system (Millipore, Bedford, MA, USA).

### 4.2 Ethical Aspects

This work was carried out in accordance with the institutional Code of Ethics. For each specimen, voucher specimens were prepared for identification and cataloging in the Herbarium of the University of Caxias do Sul (HUCS), under collection authorization SISGEN A5D3471.

Additionally, the methodological part involving the cytotoxicity study with *A. salina* did not require approval from our university's animal ethics committee, as the cysts of this crustacean were purchased from Artemia Salina (Rio Grande do Norte, Brazil), for scientific research and fish feed purposes. Because this animal is used in a wide range of environments, our institution's ethics committee has no rules regarding its use. However, we declare that this work was conducted in accordance with the Code of Ethics for the Treatment of Animals. The manuscript complies with the Guidelines for Conduct in Reporting, Editing, and Publication of Academic Articles in Medical Journals. We ensured that our conduct complied with relevant laws and guidelines.

### 4.3 Plant Material

Ten NCFPs were collected in southern Brazil (state of Rio Grande do Sul, within the municipality of Caxias do Sul: (1) Grotão; (2) Vila Oliva; (3) Santa Lúcia do Piaí; (4) Nova Petrópolis; and (5) the Campus of the University of Caxias do Sul). The collections were carried out between March 2018 and February 2019 (corresponding to the period of highest fruiting incidence). The collected fruits were sanitized with a 1% sodium hypochlorite solution, washed three times with distilled water to ensure complete removal of chlorine residues and to prevent potential induction of toxicity, and identified. All samples were then frozen at −20°C for further processing and preparation of extracts. All NCFPs collected were identified by two researchers with experience in the field (**Researcher 1**: Bachelor's and Licentiate in Biological Sciences, Master's and PhD in Botany and Curator of the HUCS Herbarium of the University of Caxias do Sul and **Researcher 2** ‐ principal researcher and correspondent of this manuscript—Bachelor's and Licentiate in Biological Sciences, Master's in Biotechnology, PhD in Biotechnological Processes and Professor in the area of ​​Botany, Agroecosystem Ecology and Agroecology of the University of Caxias do Sul). These plants are stored in the Herbarium of the University of Caxias do Sul and listed in Table 3.

**TABLE 3:** Botanical characterization and descriptive data of NCFPs collected in southern Brazil.

Scientific name	Commom name	Botanical family	Herbarium record
*Citharexylum solanaceum* Cham	Tarumã	Verbenaceae	47153
*Ilex theezans* Mart. ex Reissek	Caúna	Aquifoliaceae	47151
*Myrceugenia miersiana* (Gardner) Legrand & Kausel	Araçarana	Myrtaceae	49777
*Myrcia oblongata* DC	Guamirim	Myrtaceae	47148
*Myrsine parvula* (Mez) Otegui	Capororoca	Primulaceae	49331
*Psidium cattleyanum* Sabine	Araçá‐vermelho	Myrtaceae	50848
*Solanum pseudoquina* A. St.‐Hill	Joá‐de‐árvore	Solanaceae	47149
*Tournefortia paniculata* Vent.	Marmelinho	Boraginaceae	49351
*Vitex megapotamica* (Spreng.) Moldenke	Tarumã	Lamiaceae	50836
*Zanthoxylum rhoifolium* Lam.	Mamica de cadela	Rutaceae	47150

### 4.4 Processing and Obtaining Extracts

The collected fruits were crushed in a mixer (Philco PMX2000 3 in 1 Inox 800 W) until a homogeneous paste was obtained. The extracts were prepared using distilled water at a concentration of 5% (w/v) and boiled at 100°C for 15 min in a reflux system. Subsequently, they were filtered in a vacuum pump (Fisatom 820, 40 MBAR—Biotecmed) and frozen under protection from light for future analysis. All extracts were subjected to antioxidant and toxicity assays immediately after preparation, without prior storage. For potential subsequent analyses, aliquots were stored frozen under light‐protected conditions until use [68].

### 4.5 Determination of the Antioxidant Capacity of Extracts

The antioxidant activity was determined by the DPPH (2,2‐diphenyl‐1‐picrylhydrazyl) free radical scavenging capacity method. Thus, 100 µL of each extract was added to 500 µL of DPPH reagent and 400 µL of 100 mM Tris‐HCl buffer (pH 7.0). The samples were shaken and then left to rest and protected from light for 20 min. Afterwards, the absorbances were determined at 517 nm, in a spectrophotometer (UV‐1700, Shimadzu Pharmaspec). The results were expressed as a percentage of inhibition of the DPPH free radical [69].

The ability of the extracts to reduce the ABTS radical was verified by reacting with an aqueous solution of ABTS (7 mM) with a solution of potassium persulfate (140 mM). This solution was kept in the dark at room temperature for 12–16 h before use. Then, the ABTS^+^ solution was diluted with ethanol (99%) to an absorbance of 0.700 ± 0.02 at 734 nm. Then, 1.0 mL of diluted ABTS solution was added to 10 µl of the different extracts evaluated. The absorbance in a spectrophotometer (UV‐1700, Shimadzu Pharmaspec) was taken exactly 6 min after the initial mixing. The results were expressed as % inhibition of the ABTS radical [70].

### 4.6 Total Phenolic Content of Extracts—Folin‐Ciocalteu

To this assay, 100 µL of sample was added to 500 µL of 1 N Folin‐Ciocalteu reagent and 400 µL of 7.5% calcium carbonate (Na_2_CO_3_). For the standard curve, gallic acid was used in concentrations ranging from 0.025 to 0.75 mg/mL. The samples were shaken and remained at rest and protected from light for 30 min. Absorbances were determined at 765 nm in a spectrophotometer (UV‐1700, Shimadzu Pharmaspec). The results were expressed in mg of gallic acid equivalents (GAE)/g^−1^ of extract [71].

### 4.7 Quantification and Characterization of Major Phenolic Compounds—Validation of the Analytical Method

The analytical methodology was validated by evaluating selectivity, linearity, and precision. The detection and quantification limits were also calculated [72]. Selectivity was verified by comparing the retention times of the peaks of the standard compounds. Linearity was analyzed with analytical curves of each standard in triplicate, at six different concentrations [72]. Precision was determined by the repeatability of the analysis of the same sample in nine replicates, with the results expressed as the mean and relative standard deviation. The limits of detection (LD) and quantification (LQ) were calculated by dividing the linear coefficient by the angle of the analytical curves and multiplying the results by 3 for LD and by 10 for LQ [72].

### 4.8 Analysis of Phenolic Compounds by High‐Performance Liquid Chromatography—HPLC‐UV

The extracts of NCFP were analyzed in HPLC equipment (HP model 1100), Lichrospher RP18 column (5 µm) coupled to a UV detector at 210 nm (HPLC‐UV) and a quaternary pump system. The reversed phase analysis consisted of solvent A—Milli‐Q water with 1% phosphoric acid, and solvent B—Acetonitrile. The mobile phase pumping system was a gradient, with 90% solvent A from 0 to 5 min, 60% A from 5 to 40 min, and 90% A from 45 to 50 min. The standard flow was maintained at 0.5 mL/min. The injection volume was 10 µL. The samples were filtered through Nylon membranes with a 0.45 µm pore diameter. Phenolic compounds were identified according to their elution order and by comparing their retention time with those of their pure standards. Quantification was performed by the external standardization method, by correlating the area (mAU*s) of the compound peak to the standard curve performed with each standard evaluated: gallic acid, epigallocatechin, catechin, epicatechin, epigallocatechin gallate, rutin, vitexin, ferulic acid, naringin, hesperidin, myricetin, resveratrol, quercetin, and apigenin [73].

### 4.9 Evaluation of in Vivo Toxicity in *Artemia salina*

The acute toxicity of the extracts was evaluated by incubating eggs *A. salina* (0.2 g—*A. salina* cysts were purchased from Artêmia Salina, Rio Grande do Norte, Brazil) in a rectangular aquarium (20 × 15 × 10 cm) containing 2 L of artificial saline water (distilled water added with 36.5 g.L^−1^ of sea salt) at controlled pH (7.0 ± 2) with controlled artificial light and temperature (27 ± 3°C). A 60‐W incandescent lamp provided heating and lighting. After 30 h of incubation, 0.2 g of dry biological yeast (*Saccharomyces cerevisiae*) was added as a food source for the nauplii. After 48 h, 18 to 22 nauplii were transferred to 24‐well plates containing 0.8 mL of saline solution. Then, 0.2 mL of the extract solutions at different concentrations was added. The tested extract concentrations ranged from 1 µg mL^−1^ to 5000 µg mL^−1^. A pure saline solution was used as the negative control, while varying concentrations of potassium dichromate (100, 75, 50, 25, 10, 5, and 1 µg mL) served as the positive control. The plates were kept for 24 h under the environmental conditions of the incubation system. The analysis was performed using a colony counter (CP 608, Phoenix). Nauplii that were immobile after light stimulation were considered dead. The LD_50_ values ​​were obtained by interpolating 50% (*y* = 50) of the dead ones in the trend curves [74].

### 4.10 Statistical Analysis

All analyses were performed in triplicate, and the results were expressed as mean and standard deviation. Data were checked for normality by Kolmogorov‐Smirnov, and the results were subjected to analysis of variance (ANOVA) followed by Tukey's post hoc test. Pearson's correlation coefficient was used to correlate antioxidant activity and total phenolic compound content. Statistical significance was set at *p* < 0.05, using SPSS software version 22.0 (SPSS, Chicago, IL).

## Author Contributions

Luana Vanessa Peretti Minello, Paola Dutra da Rosa and Sabrina dos Santos Cassol: conducted the experiments. Luciana Bavaresco Andrade Touguinha, Cátia Santos Branco and Valdirene Camatti Sartori: conceived the experiments and analyzed the results. All authors read and approved the manuscript.

## Funding

This study did not receive any type of funding. Scholarship provided by the Coordination for the Improvement of Higher Education Personnel—Brazil (CAPES) with code 001.

## Conflicts of Interest

The authors declare no conflict of interest.

## Data Availability

The data supporting the findings of this study are available from the corresponding author upon reasonable request.
